# Emodin Inhibits Homocysteine-Induced C-Reactive Protein Generation in Vascular Smooth Muscle Cells by Regulating PPARγ Expression and ROS-ERK1/2/p38 Signal Pathway

**DOI:** 10.1371/journal.pone.0131295

**Published:** 2015-07-01

**Authors:** Xiaoming Pang, Juntian Liu, Yuxia Li, Jingjing Zhao, Xiaolu Zhang

**Affiliations:** 1 Department of Pharmacology, Xi’an Jiaotong University School of Medicine, Xi’an, China; 2 Department of Clinical Pharmacy, Central Hospital of Zibo, Zibo, China; University of Louisville, UNITED STATES

## Abstract

Atherosclerosis is an inflammatory disease. As an inflammatory molecule, C-reactive protein (CRP) plays a direct role in atherogenesis. It is known that the elevated plasma homocysteine (Hcy) level is an independent risk factor for atherosclerosis. We previously reported that Hcy produces a pro-inflammatory effect by inducing CRP expression in vascular smooth muscle cells (VSMCs). In the present study, we observed effect of emodin on Hcy-induced CRP expression in rat VSMCs and molecular mechanisms. The *in vitro* results showed that pretreatment of VSMCs with emodin inhibited Hcy-induced mRNA and protein expression of CRP in a concentration-dependent manner. The *in vivo* experiments displayed that emodin not only inhibited CRP expression in the vessel walls in mRNA and protein levels, but also reduced the circulating CRP level in hyperhomocysteinemic rats. Further study revealed that emodin diminished Hcy-stimulated generation of reactive oxygen species (ROS), attenuated Hcy-activated phosphorylation of ERK1/2 and p38, and upregulated Hcy-inhibited expression of peroxisome proliferator-activated receptor gamma (PPARγ) in VSMCs. These demonstrate that emodin is able to inhibit Hcy-induced CRP generation in VSMCs, which is related to interfering with ROS-ERK1/2/p38 signal pathway and upregulating PPARγ expression. The present study provides new evidence for the anti-inflammatory and anti-atherosclerotic effects of emodin.

## Introduction

Cardiovascular diseases resulting from atherosclerosis are the leading cause of mortality and morbidity all over the world. In traditional views, atherosclerosis is considered as a progressive narrowing of the artery lumen due to hyperlipidemia. Nevertheless, epidemiological analysis reveals that atherosclerosis still occurs in patients with normal or low cholesterol level [1]. In the recent years, a growing number of evidence from laboratory and clinical studies show that inflammation plays a pivotal role in all phases of atherogenesis, and therefore support that atherosclerosis is a chronic inflammatory disease [2,3].

Among the various inflammatory molecules, C-reactive protein (CRP) is a representative inflammatory cytokine. CRP is not only considered as the most reproducible marker to predict cardiovascular risk, but also accepted as an independent risk factor for cardiovascular events [4]. Moreover, an increasing number of data suggest that CRP directly participates in the initiation and progression of atherosclerosis through multiple activities in the inflammatory response [5].

Elevated plasma homocysteine (Hcy) level is an independent risk factor for atherosclerosis [6, 7], and hyperhomocysteinemia is estimated to account for 10% of cardiovascular events [8]. Data from cell culture, animal experiments and clinical investigations suggest that Hcy appears to promote atherosclerosis formation by the pleiotropic effects including pro-inflammation [9]. Our previous research confirmed that Hcy is able to stimulate CRP expression in rat vascular smooth muscle cells (VSMCs) via reactive oxygen species (ROS) and mitogen activated protein kinase (MAPK) signal pathway [10]. Moreover, the locally generated CRP is anticipated to intensify the inflammatory process in the vessel wall and thus contribute to atherogenesis [11].

Emodin, 1, 3, 8-trihydroxy-6-methylanthraquinone, is a natural active ingredient found in many Chinese herbs, such as *Rheum officinale*, *Rheum palmatum* and *Polygonam cuspidatum*. In addition to its traditional usage as a laxative, emodin exhibits various biological activities including anti-cancer, anti-bacterial, anti-oxidant, and anti-inflammatory effects [12]. Meng *et al*. report that emodin inhibits TNF-α-induced expressions of MMP-2 and MMP-9 in VSMCs to produce an anti-inflammatory effect [13]. Despite the report, there is no direct evidence to prove that emodin exerts an anti-inflammatory effect through inhibition of CRP production in vascular cells. Therefore, the present study observed the inhibitory effect of emodin on Hcy-stimulated CRP expression in VSMCs, and probed the roles of ROS-MAPK signal pathway and peroxisome proliferator-activated receptor gamma (PPARγ) in the effect so as to provide a new evidence for its anti-inflammatory and anti-atherosclerotic effects.

## Materials and Methods

### Reagents

Emodin, isolated from the root of *Rheum officinale Baill*, was provided by Shaanxi Zhongxin Biotechnology Co. Ltd (Xi’an, China) and dissolved in dimethyl sulfoxide (DMSO) for use. DL-homocysteine and 3-(4,5-dimethylthiazol-2-yl)-2,5-diphenyltetrazolium bromide (MTT) were from Sigma–Aldrich (St. Louis, MO, USA). Polyclonal anti-rat CRP and anti-rat PPARγ antibodies were from Abcam (Cambridge, UK). Antibodies against phospho-ERK1/2, ERK1/2, phospho-p38, p38 and 2’,7’-dichlorodihydrofluorescein diacetate (H_2_DCF-DA) were from Beyotime (Jiangsu, China). ELISA kit for detecting CRP was from Westtang (Shanghai, China).

### Culture of rat aortic smooth muscle cells

VSMCs derived from the thoracic aorta of male Sprague-Dawley (SD) rats according to Hadrava’s method[14] were cultured in DMEM supplemented with 100 U/ml penicillin, 100 U/ml streptomycin and 10% fetal bovine serum (FBS)in the condition of 5% CO_2_ and 95% air at 37°C. The cells between passages 3 and 8 were used in the experiments. Before the experiments, the cells was starved for 24 h in DMEM containing 0.1% FBS. All the experimental procedures were approved by the Institutional Animal Care Committee of Xi’an Jiaotong University.

### Assessment of the cell viability

The viability of VSMCs was examined by the MTT method [10]. VSMCs seeded in a 96-well plate at a concentration of 10^4^ cells/well were incubated for 24 h with 0.1, 1, 10, 100 μM emodin or DMEM for control. Then, 20 μl MTT (5 mg/ml) was added to each well for 4 h’s further incubation at 37°C. After that, the culture medium was removed and the formazan crystal was completely dissolved by 150 μl DMSO. Finally, formazan absorbance was assessed at 490 nm with an ELISA-reader (Thermo, NH, USA)

### Reverse transcription polymerase chain reaction (RT-PCR)

The total cellular RNA from VSMCs was purified with an RNA purification kit (Fastagen, Shanghai, China). Complementary DNA was synthesized using oligo (dT)18 primer and M-MuLV reverse transcriptase (Fermentas, St. Leon-Rot, Germany). Primers for CRP (sense primer: 5'-CATCTGTGCCACCTGGGAGTC-3', antisense primer: 5'-AAGCCACCGCCATACGAGTC-3', yielding 153 bp fragment, GenBank Accession No. NM_017096) and GAPDH (sense primer: 5'-GCAAGTTCAACGGCACAGTCAAG-3', antisense primer: 5'-ACATACTCAGCACCAGCATCACC-3', yielding 124 bp fragment, GenBank Accession No. NM_017008) were synthesized by Sangon (Shanghai, China). PCR amplification was performed through 35 cycles at 94°C for 30 s, 58°C for 30 s, 72°C for 45 s, and final extension of PCR products was performed for 5 min at 72°C. PCR products were separated by electrophoresis on 2% agarose gel with ethidium bromide. Expression of mRNA was quantified as relative to internal control GAPDH.

### Western blot analysis

The treated cells were lysed by 200 μl ice-cold RIPA lysis buffer supplemented with the protease inhibitor cocktail (Roche, Mannheim, Germany). Protein concentration was measured by NanoDrop spectrophotometer (NanoDrop, Wilmington, USA). Equal amount of protein extract (50 μg) was loaded, separated by 10% SDS-PAGE, and blotted onto nitrocellulose membrane. Then, the membranes were incubated with anti-CRP (1:200 dilution), anti-PPARγ (1:500), anti-p38 (1:500 dilution), anti-phospho-p38 (1:500 dilution), anti-ERK1/2 (1:500 dilution), anti-phospho-ERK1/2 (1:500 dilution) and anti-β-actin (1:1000 dilution) antibodies overnight at 4°C. After washed, the membranes were incubated with appropriate IgG antibody conjugated with horseradish peroxidase for 1.5 h at room temperature followed by the enhanced chemiluminescence (Pierce, Rockford, USA). The light signals were detected by X-ray film. Intensity of the specific band was analyzed by densitometry with ImageJ (NIH, MD, USA). Beta-actin was used as loading control.

### Measurement of ROS

H_2_DCF-DA fluorescent probes were used to detect the intracellular ROS in VSMCs [15]. VSMCs incubated with the different concentrations of emodin (0.1, 1, 10 μM) for 24 h were exposed to Hcy (100 μM) for 40 min after preloaded with H_2_DCF-DA (10 μM) for 30 min at 37°C. The fluorescence was detected by a fluorescence microscope (Olympus BX51, Japan) at the excitation wavelength of 488 nm and the emission wavelength of 525 nm.

### Methionine diet-induced hyperhomocysteinemic model

Male SD rats aged 8 weeks from Experimental Animal Center of Xi’an Jiaotong University School of Medicine were divided into control group, hyperhomocysteinemic model group and two emodin-treated groups. Rats in control groups were maintained with normal diet, while rats in the model group and emodin-treated groups were fed with diet contained 2% methionine for 4 weeks [16]. Meanwhile, emodin was orally administrated to rats in emodin-treated groups at the dose of 40 or 80 mg/kg per day for 4 weeks. At the end of the experiment, blood samples were collected via the abdominal aorta, and serum was separated for detecting Hcy and CRP levels. Then, the thoracic aorta of rats was removed for analyzing mRNA expression of CRP with RT-PCR, and detecting protein expressions of CRP, PPARγ, ERK1/2, p38, p-ERK1/2 and p-p38 by Western blot. Hcy concentration was examined by a commercial available kit based on the enzyme cycling method (Beijing Strong Biotechnologies, Beijing, China), and CRP levels were assayed by ELISA.

### Statistical analysis

All values were shown as mean ± S.E.M. Statistical significance between groups was assessed by one-way ANOVA followed by Tukey’s test. *P* < 0.05 was considered statistically significant.

## Results

### Effect of emodin on the viability of VSMCs

**Fig 1:**
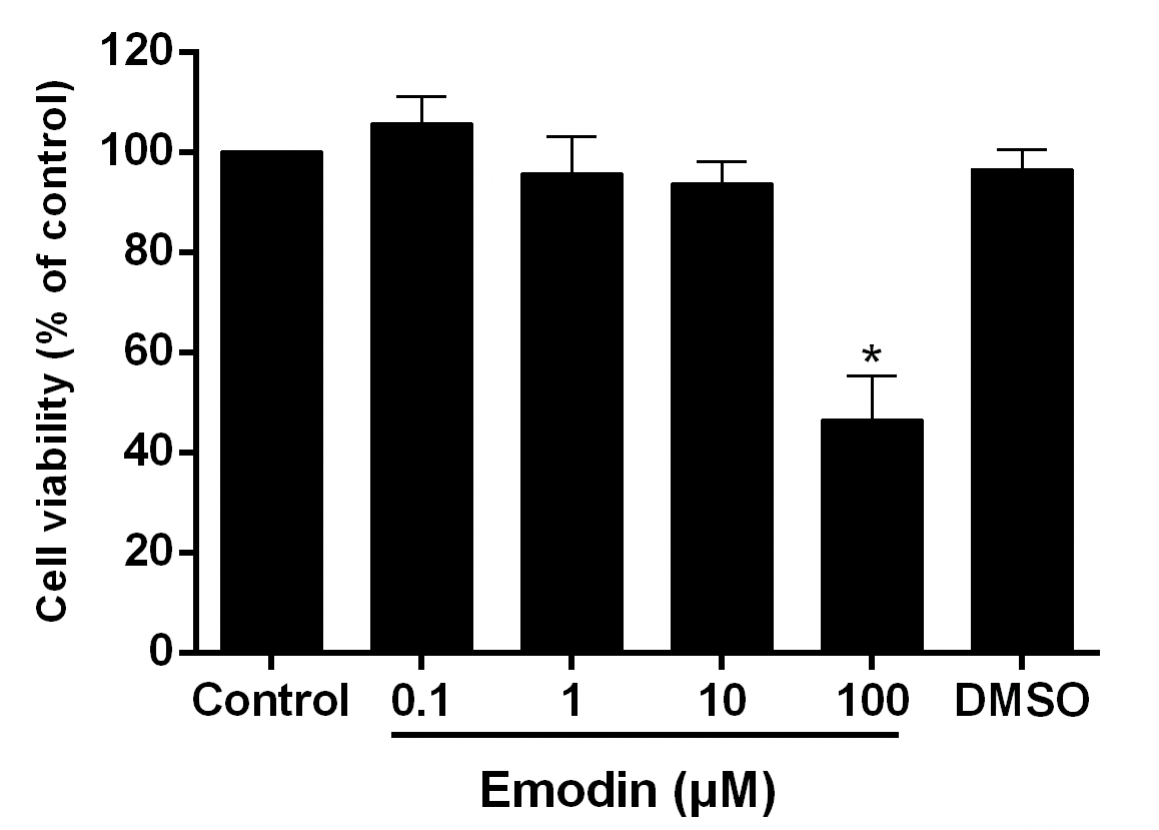
Effect of emodin on the viability of VSMCs. The cells were incubated with the different concentrations of emodin for 24 h. Then, the cell viability was assayed by the MTT method. DMSO (0.1%) was used as solvent control. Results from three independent experiments were expressed as mean ± S.E.M. *P < 0.05 *vs*. DMSO.

In order to choose the proper concentrations of emodin used for the *in vitro* study, effect of emodin on the viability of VSMCs was observed. Fig 1 displays that incubation of the cells for 24 h with emodin at 0.1 μM to 10 μM hardly affected the viability of VSMCs. But, 100 μM emodin significantly decreased the cell viability by 53.5% (*P* < 0.05 *vs*. DMSO). Therefore, 0.1, 1, 10 μM of emodin were used for the *in vitro* study.

### Effect of emodin on Hcy-induced CRP expression in VSMCs

**Fig 2:**
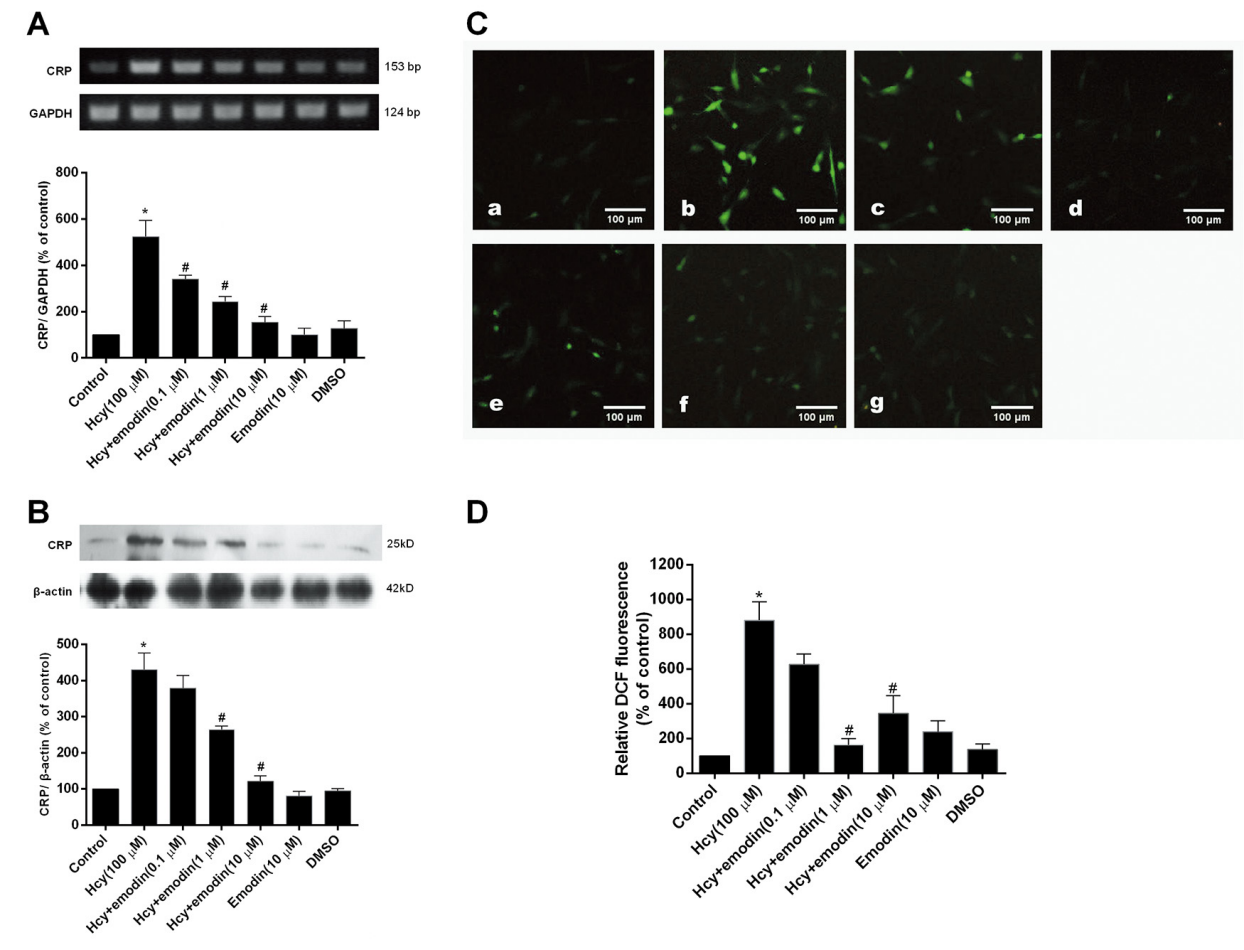
Effects of emodin on Hcy-stimulated CRP expression and ROS generation in VSMCs. The cells were preincubated with the different concentrations of emodin for 24 h before stimulation with 100 μM homocysteine (Hcy) for 12 h. mRNA (A) and protein (B) expression of CRP was identified by RT-PCR and Western blot, respectively. In another experiment, the cells were incubated with the different concentrations of emodin for 24 h and then, exposed to 100 μM Hcy for 40 min after preloaded with H_2_DCF-DA (10 μM) for 30 min. The fluorescent intensity was measured by a fluorescence microscope. (C) Representative fluorescence images: (a) control, (b) Hcy alone, (c) Hcy + 0.1 μM emodin, (d) Hcy + 1 μM emodin, (e) Hcy + 10 μM emodin, (f) 10 μM emodin, (g) 0.1% DMSO. (D) Relative fluorescence intensity quantified from the fluorescence images. Emodin alone was used as drug control and DMSO (0.1%) was used as solvent control. Results from three independent experiments were expressed as means ± S.E.M. *P < 0.05 *vs*. control, ^*#*^ *P* < 0.05 *vs*. Hcy alone.

Fig 2A and 2B show that CRP mRNA and protein expression was increased after the exposure of VSMCs to 100 μM Hcy for 12 h. (*P* < 0.05 *vs*. control). However, pretreatment of the cells with the different concentrations of emodin (0.1, 1, 10 μM) prior to Hcy stimulation markedly inhibited Hcy-induced mRNA and protein accumulation of CRP in VSMCs in a concentration-dependent manner (*P* < 0.05 *vs*. Hcy alone). Inhibition of CRP was 34.9%, 53.3%, 70.7% for mRNA expression, and 11.9%, 38.5%, 71.8% for protein expression, respectively. In addition, emodin or DMSO (solvent control) alone did not significantly change the basal CRP expression in VSMCs.

### Effect of emodin on Hcy-stimulated intracellular ROS generation in VSMCs

As shown in Fig 2C and 2D, there was a basal DCF fluorescence in control VSMCs, which represented ROS generation. Exposure of the cells to 100 μM Hcy for 40 min resulted in a significant raise of DCF fluorescence with 8.8 times over control (*P* < 0.05 *vs*. control). After preincubation of the cells with emodin at 0.1, 1 and 10 μM, Hcy-stimulated ROS generation was markedly diminished (*P* < 0.05 *vs*. Hcy alone), and inhibitory rate was 28.9%, 81.4% and 60.9% respectively.

### Effects of emodin on Hcy-regulated MAPK phosphorylation and PPARγ expression in VSMCs

**Fig 3:**
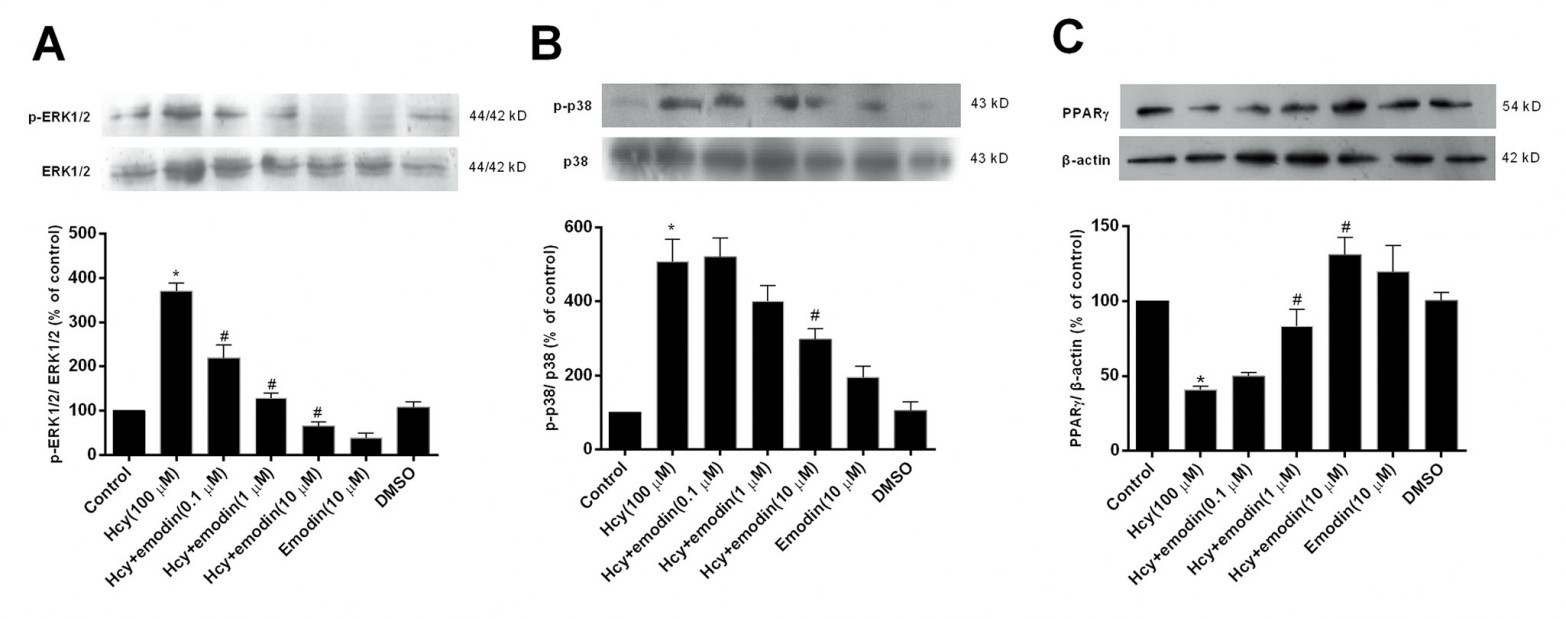
Effects of emodin on Hcy-activated ERK1/2 and p38 phosphorylation and Hcy-restrained PPARγ expression in VSMCs. After pretreatment with the different concentrations of emodin for 24 h, the cells were stimulated with 100 μM homocysteine (Hcy) for 1 h for ERK1/2 and p38 phosphorylation, and for 12 h for PPARγ expression. Then, ERK1/2 (A) and p38 (B) phosphorylation and PPARγ protein expression (C) were detected by Western blot. Emodin alone was used as drug control and DMSO (0.1%) was used as solvent control. Results from three independent experiments were expressed as means ± S.E.M. *P < 0.05 *vs*. control, #*P* < 0.05 *vs*. Hcy alone.

Our previous study found that Hcy is capable of activating ERK1/2 and p38, which mediate Hcy-induced CRP expression in VSMCs [10]. In the present study, effect of emodin on Hcy-induced activation of ERK1/2 and p38 was assessed by Western blot analysis. The results in Fig 3A and 3B illustrated that Hcy facilitated phosphorylation of ERK1/2 and p38 in VSMCs (*P* < 0.05 *vs*. control), while emodin ameliorated Hcy-activated phosphorylation of ERK1/2 and p38 (*P* < 0.05 *vs*. Hcy alone).

The result in Fig 3C revealed that incubation of the cells with 100 μM Hcy for 12 h reduced PPARγ expression (*P* < 0.05 *vs*. control). Compared with Hcy alone, pretreatment of the cells with emodin for 24 h antagonized the inhibition of PPARγ expression by Hcy in a concentration-dependent manner (*P* < 0.05 *vs*. Hcy alone).

### Effects of emodin on serum Hcy and CRP in hyperhomocysteinemic rats

**Fig 4:**
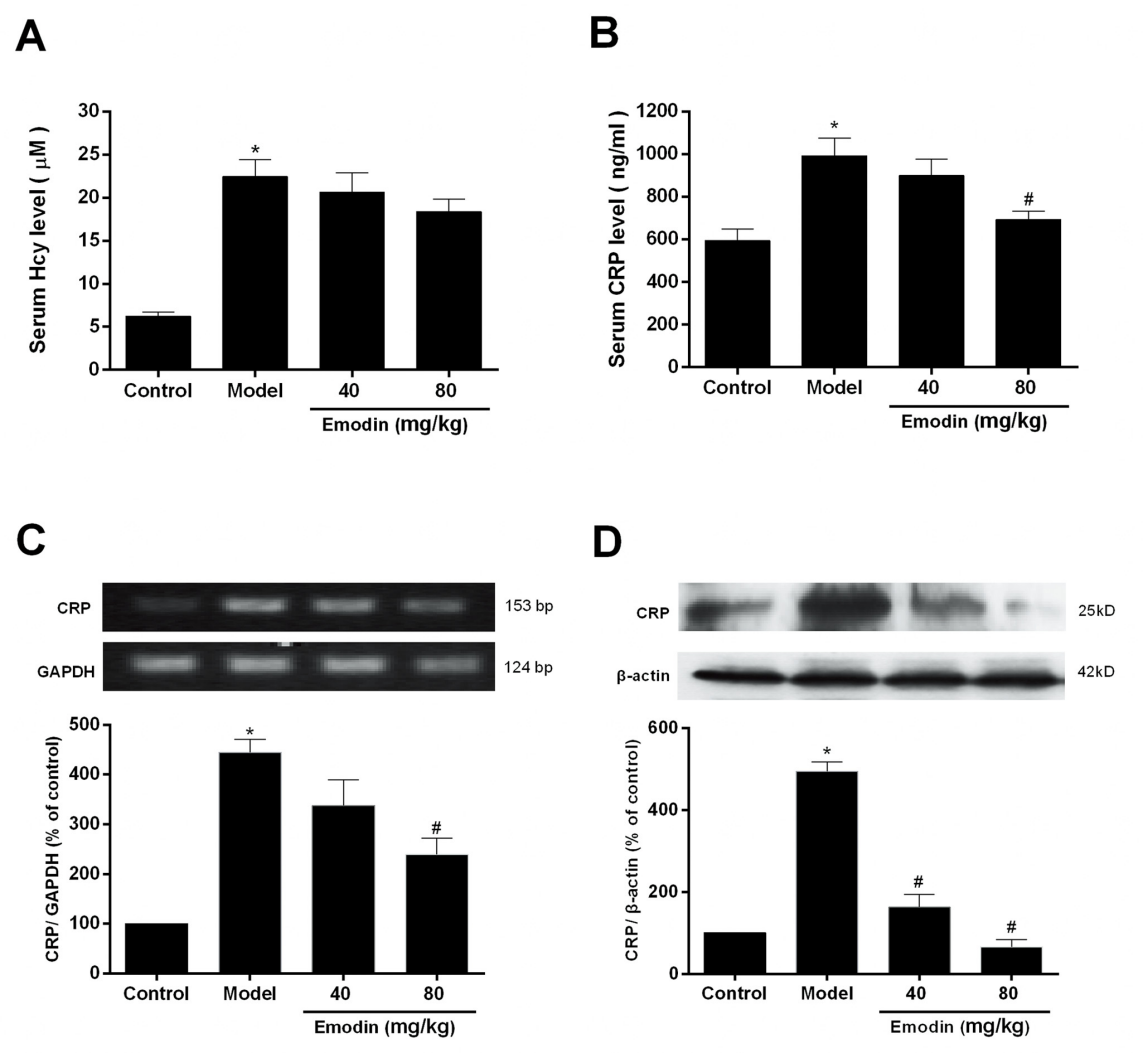
Effects of emodin on the serum Hcy and CRP levels and CRP expression in the thoracic aorta in hyperhomocysteinemic rats. Rats were fed with the high methionine diet for 4 weeks, and simultaneously treated with two doses of emodin. In the end of the experiment, the blood was collected for detecting serum Hcy level (A) with the enzyme cycling method and CRP concentration (B) with ELISA. Then, the thoracic aorta was removed for examining mRNA (C) and protein (D) expression of CRP by RT-PCR and Western blot, respectively. Data were expressed as mean ± S.E.M. (n = 8). *P < 0.05 *vs*. control, #*P* < 0.05 *vs*. model group.

As seen from Fig 4A and 4B, serum Hcy and CRP levels were significantly increased after feeding of the high methionine diet to rats for 4 weeks (*P* < 0.05 *vs*. control), whereas simultaneous administration of emodin to hyperhomocysteinemic rats decreased serum CRP concentration (*P* <0.05 *vs*. model) and but, did not change serum Hcy level.

### Effect of emodin on CRP expression in the thoracic aorta of hyperhomocysteinemic rats

Administration of the high methionine diet to rats for 4 weeks caused an obvious increase of mRNA and protein expression of CRP in the thoracic aorta (*P* < 0.05 *vs*. control). However, CRP expression was downregulated in the aortic walls of emodin-treated rats as compared with model rats (Fig 4C and 4D, *P* < 0.05 *vs*. model group).

### Effects of emodin on MAPK phosphorylation and PPARγ expression in the thoracic aorta of hyperhomocysteinemic rats

**Fig 5:**
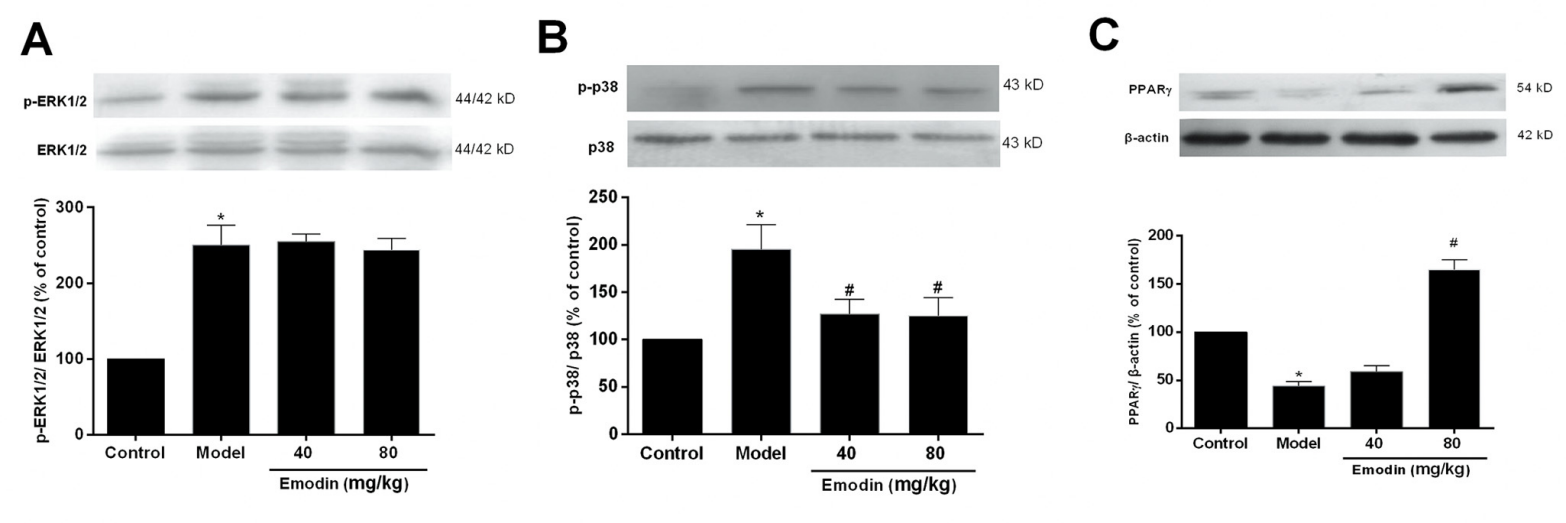
Effects of emodin on ERK1/2 and p38 phosphorylation and PPARγ expression in the thoracic aorta of hyperhomocysteinemic rats. Rats were fed with the high methionine diet for 4 weeks, and simultaneously treated with two doses of emodin. In the end of the experiment, the thoracic aorta was removed for detecting ERK1/2 and p38 phosphorylation as well as PPARγ protein expression with Western blot. Data were expressed as mean ± S.E.M. (n = 8). *P < 0.05 vs. control, #P < 0.05 vs. model group.

The results from the in vivo experiment showed that ERK1/2 and p38 phosphorylation was increased, and PPARγ protein expression was decreased in the thoracic aorta of hyperhomocysteinemic rats (*P* < 0.05 *vs*. control). Compared with model rats, p38 phosphorylation, but not ERK1/2, was decreased, and PPARγ expression was increased in the thoracic aorta of emodin-treated rats (Fig 5, *P* < 0.05 *vs*. model group).

## Discussion

It has been widely accepted that atherosclerosis is a chronic inflammatory disease in the vessel wall, and anti-inflammatory therapy is a new strategy for prevention and treatment of atherosclerosis [17]. As one of the common risk factors for cardiovascular diseases, hyperhomocysteinemia is associated with formation of atherosclerosis. We previously reported that Hcy produces a pro-inflammatory effect on VSMCs by inducing CRP expression [10]. It is known that CRP participates in initiation and progression of atherosclerosis through modulating the activities and expressions of multiple factors implicated in atherogenesis [5]. In the present study, we observed the effect of emodin on Hcy-induced CRP production in protein and mRNA levels in VSMCs. The results showed that pretreatment of the cells with emodin inhibited Hcy-induced mRNA and protein expression of CRP in a concentration-dependent manner *in vitro*. The results from the *in vivo* experiment revealed that emodin not only inhibited CRP expression in the vessel wall, but also reduced the circulating CRP level in hyperhomocysteinemic rats. These confirm that emodin is able to inhibit CRP production in VSMCs and may thus alleviate the vascular inflammation. The *in vivo* experiment also exhibited that emodin did not significantly decrease serum Hcy level of hyperhomocysteinemic rats, suggesting that the inhibitory effect of emodin on Hcy-induced CRP expression is not resulted from interfering with Hcy metabolism.

It is reported that ROS mediate Hcy-induced CRP expression in VSMCs [10], and emodin exerts an anti-oxidant property [18, 19]. The further study displayed that emodin diminished Hcy-stimulated ROS generation in VSMCs. This implicates that emodin reduces Hcy-induced CRP production possibly via inhibition of ROS generation elicited by Hcy in VSMCs.

MAPK signaling plays a pivotal role in the inflammatory process of atherosclerosis. Hcy is capable of activating MAPK [20], which is crucial for Hcy-induced CRP production in VSMCs [10]. The present results showed that emodin attenuated Hcy-activated phosphorylation of ERK1/2 and p38 in VSMCs *in vitro*, which is consistent with Zhu’s study conducted in TGF-β1-stimulated NRK-49F cells [21]. However, emodin only inhibited p38 phosphorylation, and did not significantly affect ERK phosphorylation in the thoracic aorta of hyperhomocysteinemic rats *in vivo*. The mechanism underlying the difference is unclear and merits further investigation. Existing evidence indicates that ROS serve as an upstream event of MAPK signaling, and Hcy activates ERK1/2 and p38 through stimulated ROS production in VSMCs [10]. In combination of the present results with known reports, it is inferred that emodin inhibits Hcy-activated phosphorylation of ERK1/2 and p38 via reducing ROS generation in VSMCs.

Activation of NF-κB is responsible for the expression of many inflammatory cytokines including CRP. We previously demonstrated that NF-κB plays a pivotal role in Hcy-induced CRP expression in VSMCs [10]. Furthermore, it has been demonstrated that emodin is able to inhibit the activation of NF-κB [22, 23]. Therefore, it is concluded that emodin may decrease CRP expression via inhibiting Hcy-stimulated NF-κB activation in combination of our previous results with reports from others.

PPARγ is a nuclear hormone receptor, and plays a beneficial role in the regulation of vascular inflammation [24, 25]. PPARγ agonists have been demonstrated to exert the protective effects against Hcy-mediated atherogenesis [26]. Zhou *et al*. report that emodin induces PPARγ expression in atherosclerotic plaque and promotes the plaque stability in fat-fed apolipoprotein E-deficient mice [27]. However, whether emodin regulates PPARγ expression in VSMCs remains unknown. Therefore, we observed the effect of emodin on PPARγ expression in VSMCs. The results found that emodin upregulated Hcy-inhibited PPARγ expression in VSMCs *in vivo* and *in vitro*, implicating that emodin also exerts an anti-inflammatory effect by PPARγ. However, more experiments are needed to clarify how emodin upregulates PPARγ expression and the crosstalk between PPARγ and ROS-MAPK signal pathway.

## Conclusion

The present study demonstrates that emodin is able to inhibit Hcy-induced CRP generation in VSMCs, which is related to interfering with ROS-ERK1/2/p38 signal pathway and upregulating PPARγ expression. These provide new evidence for the anti-inflammatory and anti-atherosclerotic effects of emodin.
